# A practical guide to FAIR data management in the age of multi-OMICS and AI

**DOI:** 10.3389/fimmu.2024.1439434

**Published:** 2025-01-20

**Authors:** Douaa Mugahid, Jared Lyon, Charlie Demurjian, Nathan Eolin, Charlie Whittaker, Mark Godek, Douglas Lauffenburger, Sarah Fortune, Stuart Levine

**Affiliations:** ^1^ Department of Immunology and Infectious Diseases, T.H. Chan School of Public Health, Harvard University, Boston, MA, United States; ^2^ BioMicro Center, Massachusetts Institute of Technology, Cambridge, MA, United States; ^3^ Ragon Institute of Massachusetts General Hospital (MGH), Massachusetts Institute of Technology (MIT), and Harvard, Cambridge, MA, United States; ^4^ Department of Biological Engineering, Massachusetts Institute of Technology, Cambridge, MA, United States

**Keywords:** FAIR data, systems biology, immunology, OMICs, multi-modal data, artificial intelligence, modeling, Science administration

## Abstract

Multi-cellular biological systems, including the immune system, are highly complex, dynamic, and adaptable. Systems biologists aim to understand such complexity at a quantitative level. However, these ambitious efforts are often limited by access to a variety of high-density intra-, extra- and multi-cellular measurements resolved in time and space and across a variety of perturbations. The advent of automation, OMICs and single-cell technologies now allows high dimensional multi-modal data acquisition from the same biological samples multiplexed at scale (multi-OMICs). As a result, systems biologists -theoretically- have access to more data than ever. However, the mathematical frameworks and computational tools needed to analyze and interpret such data are often still nascent, limiting the biological insights that can be obtained without years of computational method development and validation. More pressingly, much of the data sits in silos in formats that are incomprehensible to other scientists or machines limiting its value to the vaster scientific community, especially the computational biologists tasked with analyzing these vast amounts of data in more nuanced ways. With the rapid development and increasing interest in using artificial intelligence (AI) for the life sciences, improving how biologic data is organized and shared is more pressing than ever for scientific progress. Here, we outline a practical approach to multi-modal data management and FAIR sharing, which are in line with the latest US and EU funders’ data sharing policies. This framework can help extend the longevity and utility of data by allowing facile use and reuse, accelerating scientific discovery in the biomedical sciences.

## Introduction

Data powers our understanding of the world around us. As the world becomes fully digitized and technology continues to develop, researchers’ ability to gather different types of measurements at scale is only increasing, making the adoption of Data Science principles across all disciplines increasingly necessary. This is particularly true in biomedical research, where the race to understand the basis of life and human disease has encouraged researchers to push the boundaries of method development for decades. Most notably, the advent of high-throughput technologies such as high-content imaging, multi-parameter flow cytometry/CyToF, microarrays, next generation sequencing, and mass spectrometry has transformed biologic research into a rich multi-modal data science.

The increase in scale across the research enterprise requires careful experimental design as well as the development of novel statistical and mathematical frameworks that can help researchers synthesize all this data into meaningful, and occasionally non-intuitive biological insights. We collectively refer to these frameworks as artificial intelligence (AI). Practically, this necessitates making the data accessible and interpretable to other researchers as well as machines in ways that allow it to be used for applications beyond its original intent. In the case of human studies, this also requires deliberate efforts to ensure the data is representative of human diversity and is well-guarded to protect individuals’ privacy and rights. Only then can society fully benefit from the richness and complexity of the data needed to enable AI-driven advances in the biomedical field for the benefit of all.

A term commonly used to refer to good data stewardship in the life sciences is FAIR data sharing (1). The term is an acronym for data that is findable, accessible, interoperable, and reusable, all features that extend the usability of data beyond the purposes it was generated for, thereby increasing its long-term impact. Here, we outline practical steps towards FAIR data-sharing practices that can improve the longevity and utility of biomedical data. The principles are applicable to any field in the Life Sciences.

## Why share data

Before ChatGPT made conversations about AI, multi-modal data, data and computational bias so mainstream (2, 3) the biomedical field had its taste of AI’s enabling potential, yet relatively little light was shed on the central role community-wide data curation and sharing played in enabling these biomedical breakthroughs. We discuss two prominent examples below.

### AlphaFold

In the case of AlphaFold (4), which won its developers the Lasker Award in 2023 (5) and Nobel Prize in 2024 (6), years of researchers publicly depositing experimentally determined protein structures and genomic sequences was fundamental to training the underlying model (4). These data resided in public databases such as UniRef90 (7), BFD (8), Uniclust30 (9), MGnify clusters (10),and the Protein Data Bank (PDB) (11) which house some of the world’s largest collections of biologic sequences and structures, respectively. Since models are only as good as the data they were trained on, it is no surprise that proteins poorly predicted by AlphaFold are often classes that are underrepresented in nature either because they lack homologues such as orphan proteins (12), or because they are highly variable such as antibodies (13). Since its release, over a million AlphaFold predicted protein structures have been shared in the public domain (14), including the proteins of bacteria rapidly developing antibiotic resistance, thereby posing an urgent threat to global health. The entirety of the AlphaFold model is also available publicly for others to explore and expand (15), which has enabled researchers to adopt it for their use-case of choice expanding its impact even further (16). Without publicly deposited data the development of AlphaFold would not have been possible, neither would much of the science enabled by it.

### NextStrain

As part of the global COVID19 response researchers worldwide rushed to sequence and share the SARS-CoV2 genomes they isolated. SARS-CoV2 genomic sequences were centralized in a repository called NextStrain (17), which also provided researchers the world over with tools that allowed tracking mutations in the viral genome, some of which threatened to be associated with changes in transmissibility, virulence, and/or clinical presentation thereby informing public health responses (18–21). Other researchers got straight to developing vaccines against the devastating virus (22). NextStrain’s developers’ focus on data and code sharing was central to its broad utility during the COVID19 pandemic, which was accompanied by an exponential increase in the number of citations from 19 in 2018 to over 2500 citations by May 2024. Its user-friendliness and transparency likely played a role in it featuring in several policy reports (18, 19, 21).

These two examples demonstrate how data and code sharing is fundamental to driving impactful scientific advances in the digital age. Both efforts required the development of globally accessible platforms that allowed the sharing of important, standardized biomedical data at scale. This enabled others to develop computational methods that could crunch through the massive volumes of data thereby providing more researchers with usable information on which to build. As more types of data are standardized and shared, we can only begin to imagine the scope and impact of future breakthroughs.

### The shifts in funding agency requirements

While the utility of sharing SARS-CoV2 genomic data was likely obvious to many, it is difficult to imagine that the developers of PDB or NCBI predicted the development of AlphaFold. It’s even more difficult to believe that every researcher depositing their protein structures since 1971 or sequences since 1982 understood they would be individual contributors to such a development many years down the line. Even if they did, it is no secret that the current scientific eco-system lacks a short-term mechanism for rewarding raw data sharing, which is a time-consuming and laborious process many researchers find unpleasant.

Incentive systems that reward data sharing are still under development but should be possible with the popularization of digital object identifiers (DOIs) (23), which allows users to uniquely cite papers as well as code and data in the digital sphere. In the meantime, science policy makers and funders seeking to maximize return on their and/or the public’s investment in basic research have resorted to mandating data sharing. In the absence of clear mechanisms for accountability much of the enforcement currently falls to publishers. As a result, the emerging practice is for researchers to only share positive data or data that is included in a publication which means a lot of data remains unaccounted for. Another important implication is that AI algorithms are being trained disproportionately on positive data which will affect their performance and generalizability (24). That said, enforcement at publication has served science well and is arguably one of the main reasons PDB is now populated with close to 200,000 experimentally validated protein structures. Among the funders now encouraging data and code deposition are the NIH (25) and NSF (26) which are currently updating their policies following mandates from the Whitehouse Office of Science and Technology (27), as well as philanthropic organizations such as the Bill and Melinda Gates Foundation (28), and the Chan Zuckerberg Initiative (29) in the USA. In Europe, the Wellcome Trust (30) and Horizon Europe (31) mandate FAIR data sharing whenever possible.

## Key elements for responsible data use and informed reuse

### Rich metadata provides necessary context

**Figure 1:**
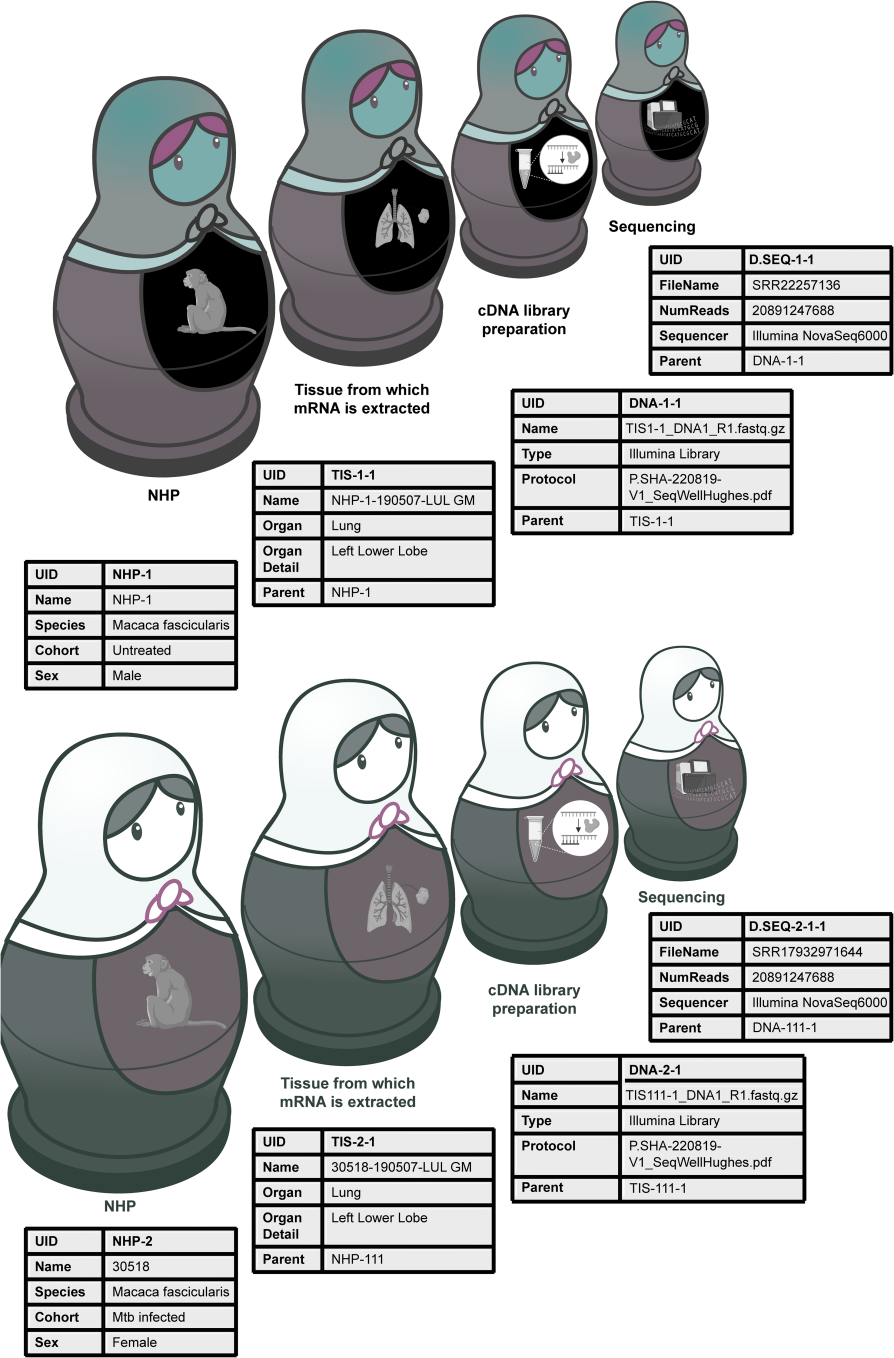
Relational databases help link metadata from multi-step experiments. Relational databases are similar to Russian dolls, nested in a particular order. They allow researchers to capture metadata at every experimental step. For example, linking sequencing data to the cDNA library, tissue (lung biopsy) and animal (non-human primate, NHP) from which it came from. The metadata for the two different sequencing files helps future users realize that the main difference between the sequenced samples is that they come from different animals that vary by sex and treatments, despite being from the same species.

Experimental data are most reusable when associated with rich metadata, often referred to as data about data, that help future users interpret and differentiate between data sets and individual data points. Imagine a set of hand-made Russian dolls. The smallest doll (the dataset) is nested within other dolls (the layers of metadata collected at each experimental step leading up to the data). Being hand-made, the innermost doll from a single set is made to fit the outer dolls perfectly, but might not fit within another set of Russian dolls, even if they were made by the same craftsperson, and even less so if made by another. If the craftsperson were to share the dimensions of each of the dolls, however, they could help others predict which inner dolls are combinable between sets (**Figure 1**). The more detailed and understandable the dimensions, the better the predicted fit, thus the need for rich and standardized metadata. In a research setting, capturing rich and understandable metadata is useful not only for researchers in the same lab, but also when re-used by others once the data is in the public domain, and is key to interoperability. Technical and biological confounders can often skew the interpretation of data and can only be accounted for if they are reported as part of the original study. This practice minimizes the chances that others will re-use the data under false assumptions leading to the generation of poorly informed hypotheses. The result is an avoidable loss of time, energy, and resources of many chasing the wrong ideas. More importantly, this contributes to the safe and cost-effective development of safer medicines for patients if such data is ever to be used in that context.

As an example, consider DNA sequencing data from a series of different tissue biopsies from a non-human primate. Each raw sequencing file is linked to a DNA library, prepared from a tissue, extracted from an animal. At the time the animal is taken into a study, it’s important to assign it a unique identifier, and document its age, sex, species and geographical origins, any interventions it underwent and when, as well as the organ from which the sample was extracted, plus the time and method of extraction. Each biopsy should also receive a unique identifier and be linked to the parent animal. Similarly, the DNA library should also receive a unique identifier and be linked to the biopsy from which it was prepared, together with information about the DNA extraction kit/process (eg: the thermocycler used, the number of amplification cycles, the temperature at each step, and the sequencing primers). Finally, it’s also important to note which samples were multiplexed on which chip (which should also have unique identifiers), the sequencer used, read length, and sequencing depth. If a plate-based fluorescence assay was done on cells from the same tissue, which sample was in which well, what was the analyte, antibody, and fluorophore, which plate reader was used, on which day, and what were the excitation and emission spectra. Much of this information can be captured in independent tables that serve as templates for researchers at every experimental step and can later be linked together as a set of relational databases, a simple but elegant and well-established solution in Data Science, that ensures full data provenance for single or multi-modal datasets from the same experiment. The metadata can then be shared in the public domain making the associated data truly FAIR (**Figure 1**).

**Figure 2:**
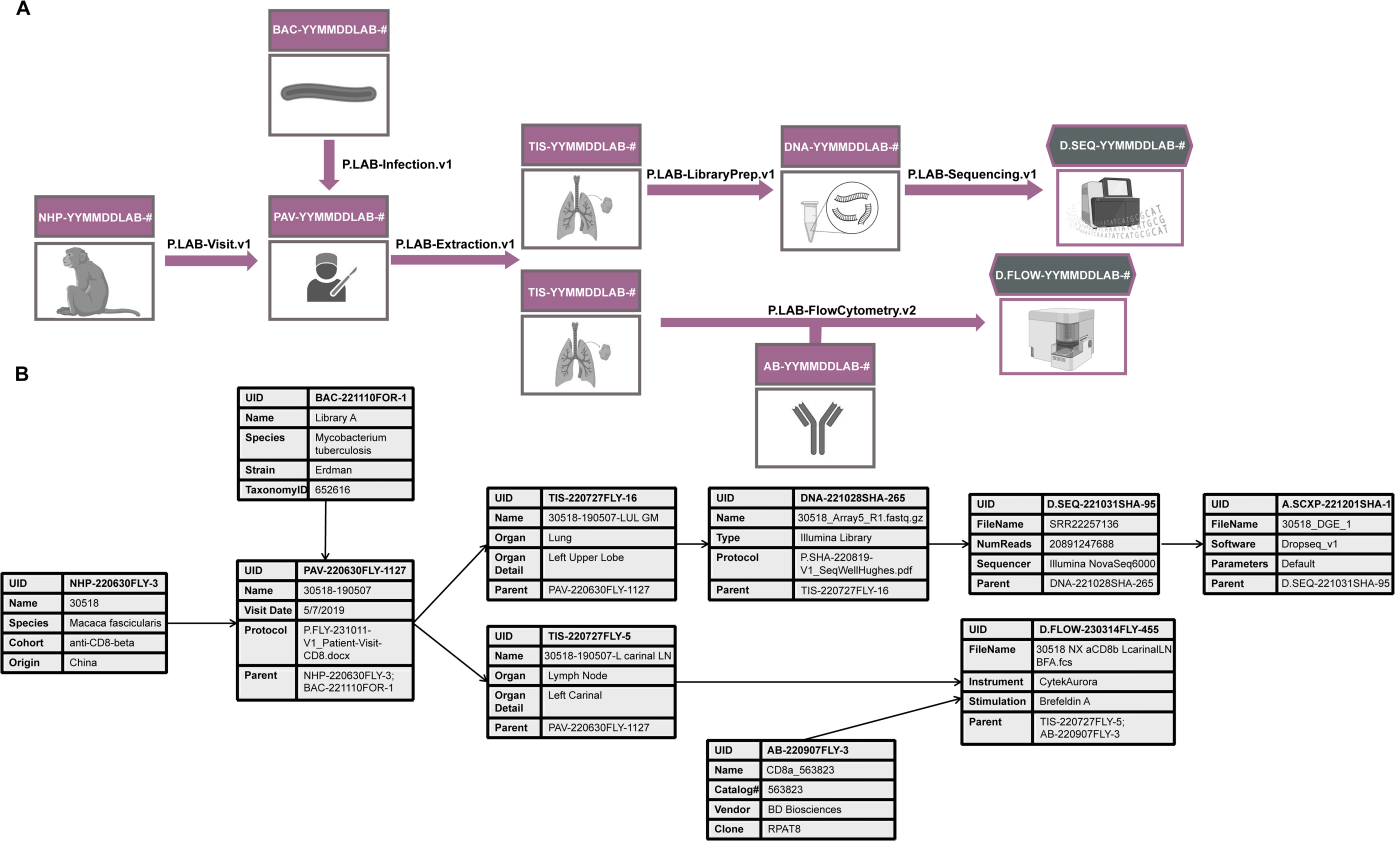
An example of how relational databases can be used to collect metadata for multi-modal data generation. **(A)** An experiment in which a lung biopsy (TIS) from a non-human primate (NHP) infected with Mycobacterium tuberculosis (BAC) is sequenced (D.SEQ) and analyzed by flow cytometry (D.Flow). DNA refers to the cDNA library sent for sequencing, AB refers to the antibody used in the flow cytometry analysis. The experimental protocol describing how each step was conducted is captured in a file denoted with a “P.” suffix and referenced in the Protocol metadata field. **(B)** An example of some metadata fields to be collected in association with each step of the research process. These fields are by no means comprehensive. Find more detailed metadata fields for a similar experiment at https://fairdomhub.org/studies/1134.

Historically, a lot of this metadata would simply be described to various degrees of thoroughness throughout a paper, but not directly linked to a particular raw or analyzed data file. While this practice might have been sufficient to interpret one small dataset at a time, it no longer serves biology well today, and adds uncertainty where it need not exist. That is especially true when combining different datatypes within a study (vertical or multi-modal data integration; **Figure 2**) or across studies (horizontal integration), where the statistical uncertainty associated with data from tissues from the same animal would be different from that from different animals or studies even if the animals were treated similarly.

Open-source data management systems can be found in NextSEEK (32) which allows public metadata sharing on FAIRDOMHub (33), or the Open Science Framework (34), though the latter is currently more suited for the social sciences. A similar, but more powerful alternative is Fairspace (35) provided commercially by The Hyve which supports the cancer research community’s c-Bioportal (36, 37). In principle, metadata collected as part of standard electronic notebook keeping can be easily exported when and where needed, facilitating FAIR data sharing through any of these systems.

### Standardization enables interoperability

Data integration has long been the focus of computational biologists, to various degrees of success (38–40) in part -some argue- because of a combination of poor data quality, experimental design and metadata availability (41–43). With the continuous increase in integratable data modalities of relevance to systems immunology, it is more important than ever to standardize how metadata is collected within and across experiments, and harmonize the vocabularies used for annotation. Unfortunately, this is no easy task. Agreeing on metadata standards for a new data type or experimental format often involves hours of discussion between experimental and computational biologists and should happen before data collection begins to avoid discrepancies down the line. This is especially true in the case of nascent technologies and requires an ability to foresee potential uses beyond what the data were originally planned for. Doing this on a field-wide level is even more challenging and requires strong vision and leadership.

For popular data types, specialist repositories often exist and some enforce the use of common data elements (44) (CDEs; pre-defined variables and acceptable values). However, these CDE are often different between repositories which makes vertical integration difficult and is further complicated by the fact that many siloed repositories offer no inherent way to link data from the same samples. This is also true in the case of inherently multi-modal measurements such as sequencing-based spatial transcriptomic technologies, which rely on a complementary set of sequencing and imaging data (45). In such cases, and in the absence of a repository for multi-modal data, the imaging data would be stored in one repository and the sequencing data in another, and would need to be linked through a public-facing database such as FAIRDOMHub (33) to be of future use (See **Table 1** for examples of existing repositories for spatial transcriptomic data). Furthermore, the metadata collected by repositories is often too sparse for meaningful analysis as they tend to focus on capturing common points of variation across experiments and not much of the nuance. Generalist repositories, being data agnostic by design, are even less suited for standardized metadata collection. Thus, it is left to depositors to decide what they think is important metadata to share, and to future users to harmonize across datasets which can be very difficult without prior standardization or the release of good dictionaries with every dataset.

**Table 1:** Key data repositories useful for systems immunology.

*Data Type*	Repository	Key Features	Number of datasets as of May 2024	Date of establishment
Mass spectrometry (MS)-based Proteomics	PRIDE: PRoteomics IDEntifications Database (46) (https://www.ebi.ac.uk/pride/)	Direct submission allowed, data visualization and annotation tools.	26847	2005
	MassIVE (47) (https://massive.ucsd.edu/)	Direct submission allowed, data analysis tools.	15,231	N/A
	PeptideAtlas (48) (https://peptideatlas.org/)	Curated database, no data analysis tools.	N/A	2006
	Panorma (49) (https://panoramaweb.org/)	Data from targeted proteomics experiments, direct submissions allowed, tools for designing and analyzing targeted proteomics experiments.	596	2014
	iProX (50) (https://www.iprox.cn/)	Direct submission allowed, no data analysis tools.	4792 Projects (3602 Public Projects)	2019
	JPOST (51) (https://repository.jpostdb.org/)	Direct submission allowed, no data analysis tools.	2671 projects	2017
MS-based Metabolomics	MetaboLights (52) (https://www.ebi.ac.uk/metabolights)	Direct submission allowed, no data analysis tools	1496	2012
	National Metabolomics Data Repository (NMDR; https://www.metabolomicsworkbench.org/data/DRCCDataDeposit.php)	Direct submission allowed, no data analysis tools.	2788	2020
ELISA, ELISPOT, Luminex	ImmPort (53) (https://www.immport.org/)	Immunology-focused, direct submission allowed, rich metadata in relational database, no data analysis tools.	262, 54, 61	2018
Flow Cytometry	ImmPort (53) (https://www.immport.org/)	Immunology-focused, direct submission allowed, rich metadata in relational database, no data analysis tools.	257	2018
	FlowRepository (54) (https://flowrepository.org/)	Direct submission allowed, follows MIFlowCyt standard, endorsed by International Society for Advancement of Cytometry (ISAC), no data analysis tools.	~2125	2012
Imaging	Image Data Resource (55) (IDR; https://idr.openmicroscopy.org/)	Direct submission allowed, handles variety of image types, no data analysis tools.	127 Studies	2017
	The Cell (CIL-CCDB) (56): (http://www.cellimagelibrary.org/)	Curated database, no data analysis.	57	2012
	Cancer Imaging Archive (TCIA) (57): (https://www.cancerimagingarchive.net/)	Data de-identified, allows direct submissions, no analysis tools.	N/A	2013
NGS and array data	Sequence Read Archive (58) (SRA; https://www.ncbi.nlm.nih.gov/sra)	Allows direct submissions of sequencing data, no analysis tools.	N/A	2007
	Database of Genotypes and Phenotypes (59) (dbGAP; https://www.ncbi.nlm.nih.gov/gap/)	Allows direct submissions of sequencing data, controlled access repository for human genotype/phenotype data.	309 general use studies ie: sharable according to these (60) terms and nothing else.	2006
	The Bioinformation and DNA Data Bank of Japan (61) (DDBJ; https://www.ddbj.nig.ac.jp/)	Allows direct submissions of sequencing and array data, provides advanced search functionalities and built-in analysis tools.	4,250,864,039 Sequences	1987
	European Nucleotide Archive (62) (ENA; (https://www.ebi.ac.uk/ena)	Allows direct submission of sequencing and data, no data analysis tools.	4.6 billion Sequences	1982
	Gene Expression Omnibus (63) (GEO; https://www.ncbi.nlm.nih.gov/geo/)	Allow direct submissions of sequencing and MIAME-compliant array data as well as processed data, some data analysis tools.	4348	2000
*Single Cell Sequencing*	Single Cell Portal: (https://singlecell.broadinstitute.org/)	Allows submission of sequencing and processed single cell data files, data visualization and analysis tools.	670 total studies found	2018
	Single Cell Expression Atlas (64) (https://www.ebi.ac.uk/gxa/sc/home)	Curated database, data visualization and analysis.	355	2018
*Spatial Transcriptomics*	CROST (65) (https://ngdc.cncb.ac.cn/crost/home)	Curated database, supports different technologies, rich suite of data analysis and visualization tools.	182	2024
	Spatial DB (66) (http://www.spatialomics.org/SpatialDB/)	Curated database, supports different technologies, some data analysis tools.	24	
	STOmicsDB (67) (https://db.cngb.org/stomics/)	Curated database, allows direct submission, some data visualization and analysis tools.	228	
	Spatial Omics DataBase (68) (SODB; https://gene.ai.tencent.com/SpatialOmics/)	Curated database, supports different technologies, some data visualization and analysis tools.	3145	2023
	Aquila (69) (https://aquila.cheunglab.org)	Curated database, allows direct submission, some data visualization and analysis tools.	110	2023
*Single Cell Sequencing*	Single Cell Portal (70) (https://singlecell.broadinstitute.org/)	Allows submission of sequencing-based spatial transcriptomic data, data visualization and analysis tools.	670 total studies found	2018
*Multi-modal OMICs*	Single Cell Atlas (71) (https://www.singlecellatlas.org/)	Curated database, multiple data types, data visualization and analysis.	NA	2024
*Generalist*	Zenodo – commercial (https://zenodo.org/)	50GB dataset limit, any file type, GitHub integration, DOI creation, version control, immediate release, usage statistics.	1,609 Projects	2013
	Figshare -commercial: (https://figshare.com/)	20 GB per user, any file type, DOI creation, version control, private and public release, usage statistics.	N/A	2012
	BioStudies (72) (https://www.ebi.ac.uk/biostudies/)	Allows the integration of metadata, orphan data, and data found in other EBI databases and link to a paper.	2,398,047	2015
	FAIRDOMHub (33) (https://fairdomhub.org)	Allows the integration of metadata, orphan data, and data found in other databases and link to a paper.	402 projects	2017

Data dictionaries clearly define each field as well as their possible values such that they are comprehensible to someone who was not part of the original study or is new to the field. It also facilitates standardization, which is why CDEs (44) are broadly useful. As an example, species could always be referred to by their NCBI Taxonomical ID and Latin name (73), their geographical origins by ISO 3166 codes (74), proteins by their Uniport ID (75), and antibodies by their company of source plus catalogue number, epitope, and conjugate. To avoid reinventing the wheel, researchers should reach for CDEs and standardized vocabularies in the public domain and share the ones they develop publicly for the benefit of others.

### Curation ensures data trustworthiness

Depending on the resources available, data sharing done well can be a time and labor-intensive process involving several people, especially when using infrastructure that is not built-for-purpose. Thus, it is important for researchers to focus on sharing high-quality (well annotated and from well-designed experiments) irrespective of whether their own analysis of the data supports their original hypotheses. This should be done with an eye towards enhancing reproducibility, but also the reuse of data for mechanistic modeling, machine learning (ML) and deep learning (DL) applications, which will undoubtedly increase the impact of the data on the long-term. As mentioned above, curated data should include both negative and positive data to avoid biased training datasets that do not allow the development of models that are broadly generalizable. If time is tight, researchers should prioritize multiplexed datasets (multi-parameter flow cytometry/CyToF, high-throughput sequencing of all kinds, mass spectrometry, high volume imaging data, array-based data, cytokine panels, systems serology data to name a few), which are most useful for data hungry ML/DL applications, as well as data acquired from experiments that are difficult to reproduce without an abundance of resources or access to highly specialized infrastructure.

## Code, model and parameter sharing facilitate reproducibility, interpretation, and informed reanalysis and meta analyses

Also important for reproducibility, integration, and the informed interpretation of analyzed data is capturing the complexity of data processing and computational analyses occurring post-generation of raw data. Unlike classical statistical tests familiar to many biologists [eg: the parametric and non-parametric tests pre-programmed in many available software suites such as Excel (76), Google Sheets (77), and GraphPad Prism (78)], many OMICs and most multi-OMIC analyses are far from standardized. Furthermore, compute environment, the choice of software, software version, and user-defined parameters can significantly affect the final output (1). For these reasons, researchers need to precisely document and share all aspects of a workflow including the code (including version number if using a publicly available package) and exact parameters used to analyze a particular dataset, with clear descriptions of any non-standard steps maybe as comments between blocks of code. This can be accomplished in a variety of ways but is greatly facilitated by the use of community workflows such as nf-core (79), containerized compute environments like Docker (80) and Singularity/Apptainer (81) shared in container repositories like DockerHub (82). Also useful are package and environment managers such as Bioconda (83). Jupyter (84) or Rstudio (85) notebooks shared and managed in code repositories such as Github (86) provide a method for sharing both standard and custom analyses, though this practice does not guarantee reproducibility across computing environments since Github does not enforce rigorous testing to ensure deposited packages are performant.

Researchers should also share their trained models given how time and computationally intensive this can be, in addition to the data on which they were trained to ensure full transparency and inform users’ understanding of sources of bias or underperformance. Parameterized mechanistic models can be shared on BioModels (87), while their machine and deep learning equivalents can be shared and deployed on Hugging Face (88).

## Resources that enable good FAIR data stewardship

### Infrastructure

**Figure 3:**
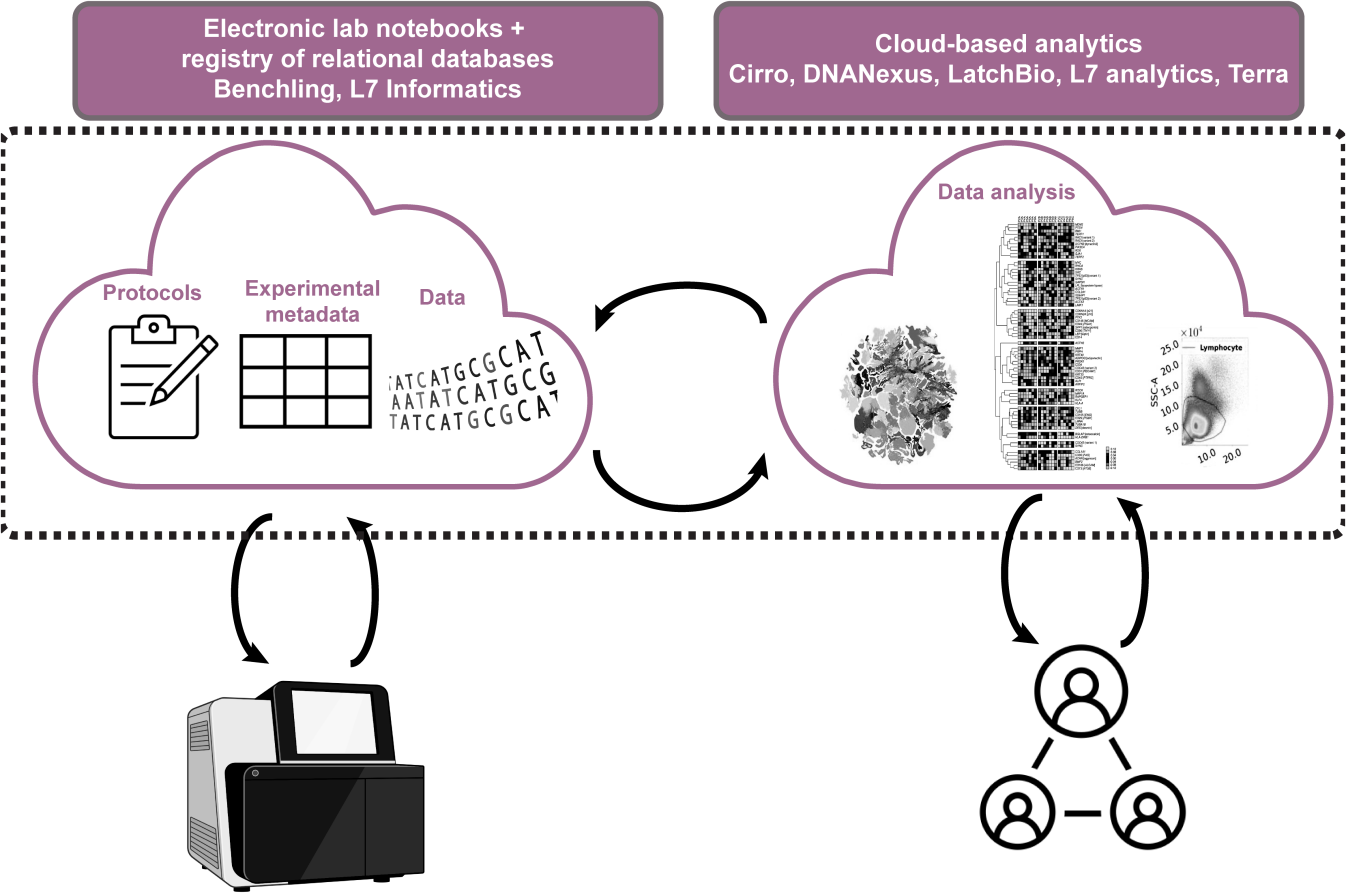
Overview of cloud-based infrastructure for digital labs with humans in the loop. Cloud-based electronic documentation systems with registries for animals, samples, reagents, equipment allow facile linking within lab notebooks. This makes experimental protocols transparent and facilitates FAIR metadata and data collection and sharing. Data from lab equipment is imported directly into the registry and can also be linked in lab notebooks saving time and effort, while minimizing human error. Metadata and data can be pushed to cloud computing platforms that allow collaborative and transparent data analysis.

To support FAIR data practices institutions must facilitate accurate data and metadata collection with little time and effort on researchers’ side. Research institutions and funders also need to account for the increasing specialization that necessitates collaboration between labs. To enable that, there is a need for a radical change in infrastructure to support an evolution to the “decentralized digital Lab with a human in the loop”. In this model, laboratory infrastructure is set up such that data acquisition and import is largely automated within and between labs with the proper agreements in place, meaning scientists spend less time generating and managing data and more time curating and analyzing it. The first step towards that has been a slow-to-start but accelerating shift from paper to electronic lab notebooks (ELNs), catalyzed by the evolution of user-friendly digital platforms such as Benchling (89). Benchling’s cloud-based ELN system now allows independent users anywhere in the world to share experimental templates, as well as track reagents, samples, and data through a shared registry, while linking these features through a set of relational databases ensuring data provenance is continuous and available to all who have access. Add to that the addition of features that allow the integration of lab instruments such that the data coming off them can be directly stored in the cloud, and the effort of moving data, linking to metadata, and -eventually- sharing no longer seems as daunting. Other providers such as L7 informatics are catching up (90). With more players in the Digital Lab eco-system the future of FAIR data sharing is looking promising (**Figure 3**). Quite importantly, this also facilitates data interpretation and saves research teams hours of lengthy discussion about how data was generated and handled.

Dedicated cloud computing platforms for biology are also emerging to compliment the shift to data-intense, decentralized and collaborative life science research, including Cirro (of the Fred Hutchinson Institute) (91), DNANexus (a techbio start up) (92), LatchBio (a techbio start up) (93), Terra (of the Broad Institute) (94), and L7 Informatics (also a techbio start up) (90). These platforms allow researchers to run complex analysis workflows in the cloud but in a more user-friendly environment than what’s offered directly by cloud providers and are customized to biologists’ needs. With the data already in the cloud, running such analyses is now possible without the need to duplicate and shuffle around large volumes of data between collaborators, and -in principle- facilitates analyses that respect institutional and national data governance requirements. Cloud providers vow they take data security seriously, and the likes of the NHS, FDA and NIH are beginning to trust them with data for megaprojects such as the UK Biobank (95) and PrecisionFDA (96) who use DNAnexus and the NIH’s All of Us Research program (97) that uses Terra (**Figure 3**). Enticingly for the computational biologists, these platforms also provide impressive compute that scales to increasingly large and complex models, come with customizable and pre-installed pipelines that save researchers hours of set-up time, and automate log generation which allows tracking the analyses done on every dataset together with the parameters used making it easy to trace how results were derived. In addition, some of these platforms support the integration of Jupyter notebooks which, as mentioned above, allow users to run and share their own custom code within those environments and share them when needed.

Less attractive is the price for cloud storage and compute which becomes an ongoing expense liable to immense runaway costs (98), especially at the hands of less experienced users who are the majority at academic institutions today. These costs could be overcome -in part- by better training, negotiating university/funder-wide contracts with cloud platforms, and could be off-set by long-term savings in personnel, maintenance, and upgrades. However, this does leave academics at the mercy of tech oligarchs such as Amazon [providers of AWS (99)], Alphabet [providers of Google Cloud (100)], and Microsoft [providers of Azure (101)]. At the moment, it is also unclear how easy migration between any of these platforms will be if they fail to meet future user needs. That said, competition in the infrastructure-as-a-service space is increasing because of ubiquitous demand across a variety of industries which will hopefully spur technological innovation and push prices down, democratizing access to infrastructure-as-a-service in the long-term.

The development of equally powerful open-source alternatives, continuously developed by and for the research community would be ideal. Unfortunately, funding such efforts is costly requiring a hefty upfront investment from governments or philanthropists and would take years adding more distance between them and their well-developed commercial counterparts. Once developed, long-term sustainability could be possible by licensing that allows free academic/non-profit usage and paid licensing in the case of for-profit entities similar to the Rosetta Commons approach (102).

### Personnel

With the emergence of infrastructure-as-a-service, better ELNs and digital lab management software (also referred to as LIMS), data, compute, and metadata are all now connectable and shareable with relative ease. But the transition to this new model is no easy feat, mostly because of the need for complex and often continuous change management since academic research inherently involves training inexperienced individuals and high turnover.

To facilitate this, the first kind of position universities need to create is that of the Data Officer. This individual outlines university-wide policy, and ensures it aligns with national and international mandates and legal frameworks. Together with the IT officer, they can help coordinate the roll out and adoption of the needed technology for the shift to Digital Labs across the university while vetting different vendors and platforms - all in coordination with Department Data Managers. The latter help roll out these changes within their departments and communicate the importance of FAIR data sharing to researchers of different disciplines, as well as work with them to develop and share best-practices that make FAIR data sharing a natural part of researchers’ workflow with the help of enabling infrastructure. They also provide guidance regarding the choice of private storage and public data repositories depending on the types of data generated (eg: sequencing, flow cytometry, imaging), its sensitivity (eg: clinical versus pre-clinical data), and its stage in the research life cycle (pre- *vs* post-publication). On the other hand, the university/department IT officers works on optimizing on premise compute and data storage requirements to adapt to a shift to the cloud. For larger, data-intensive departments, it might be necessary to have lab data managers who work even more closely with the researchers on the day-to-day. That said, buy-in from PIs is absolutely necessary for the success of these efforts and communicating the importance of good data management practices early on in every project is immensely important for labs’ long-term success. This is particularly important in academic contexts, where lab turnover is high, and data often pass multiple hands before it ends up in a paper or in the public domain.

## Common types of data and their repositories

For systems immunologists in particular and life science researchers in general, some of the most important data types today include multiplexed flow cytometry and CyTOF, Luminex, systems serology [Luminex-based antibody profiling assays (103, 104)], single cell RNASeq, bulk RNASeq data, and imaging data, for which specialized repositories currently exist. For more nascent fields or in the case of orphan datatypes researchers are encouraged to deposit their datasets in generalist repositories, until a dedicated repository is developed (**Table 1**).

## Considerations for consortia

While the guidelines outlined above are broadly generalizable, they are highly relevant for interdisciplinary academic consortia which are somewhat of a special case for three main reasons: (1) data sharing between labs in almost real-time (as opposed to at the time of publication) is important for consortia to achieve their goals as experimental and biological versus mathematical and computational expertise tend to be distributed, (2) physical samples are often exchanged between labs so tracking sample as well as data provenance at scale is key to data integrity, (3) timely exchange of knowledge requires close communication across disciplines to move project goals forward and course-correct as needed.

**Figure 4:**
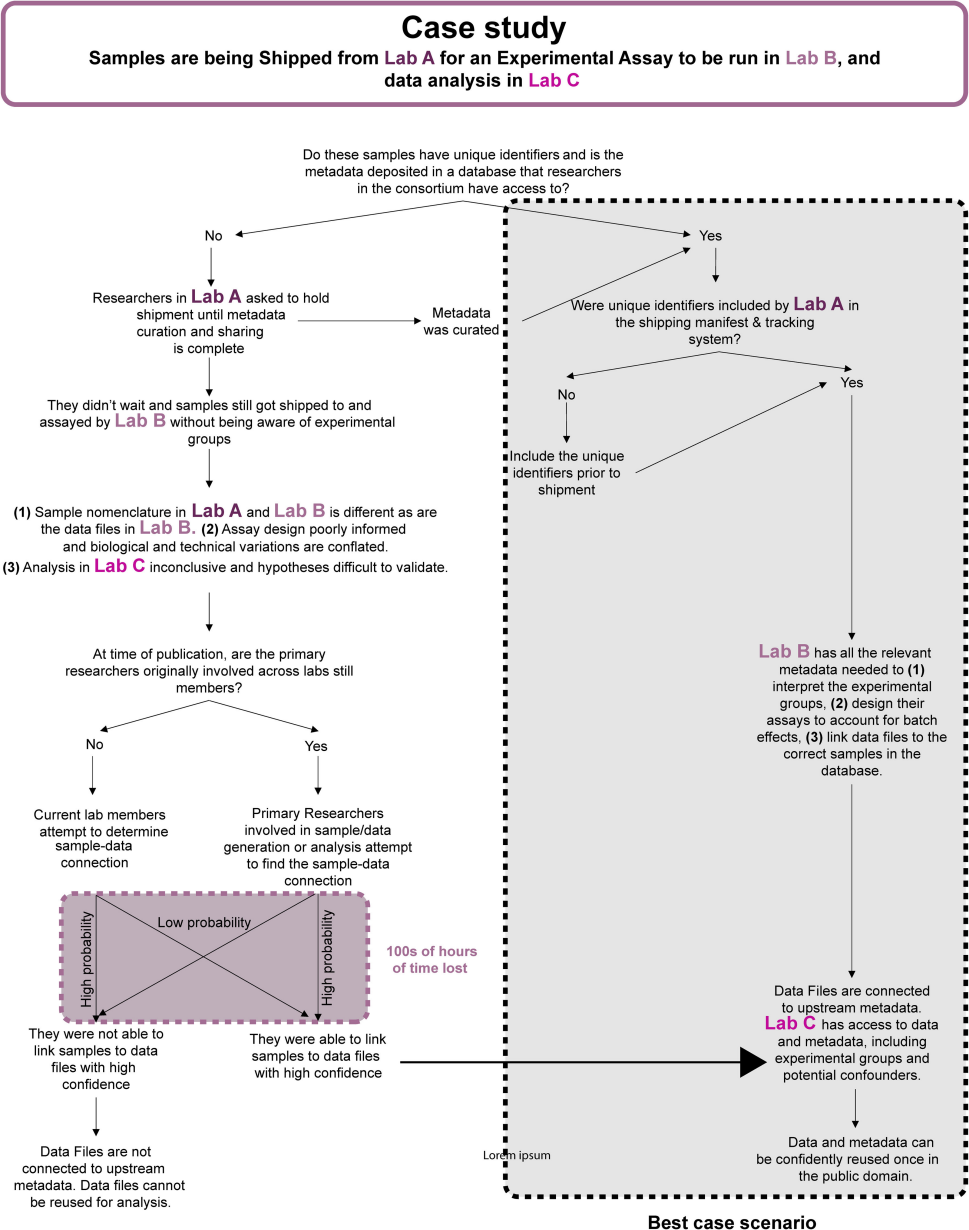
Workflow diagram describing how suboptimal practices can affect data integrity, analytical rigor, and waste valuable research time. Suboptimal practices also compromise FAIR data sharing at the end of a study.

To address the first and second point, setting up a unified or at least interoperable, digital infrastructure at the onset is key as it allows members of the consortium to share lab notebooks, reagents, and data. Harmonization and standardization becomes easier to achieve and enforce, and analyses can also be shared. This allows for internal transparency, facilitates collaboration, troubleshooting, and corrections, and ultimately multi-modal data analysis. Eventually, sharing the data and knowledge in the public domain also becomes easier (**Figure 4**).

Assigning a data officer and data base manager for the consortium is key. Together they need to establish a system that allows centralized sample and data tracking and work with each lab’s data manager and/or individual researchers to collate curated data and ensure the accompanying metadata is accurate, standardized, and complete. In instances when access to unified infrastructure is prohibitively expensive, individual components can be strung together. For example, they could set up a shared Dropbox or Google Drive account where all the consortium’s curated data is collected until it can be shared in the appropriate repositories. Setting up a local instance of NextSEEK would facilitate metadata collection in a relational database, after researchers fill out easy to use spreadsheet-based templates. Tracking samples shipped between sites can be done using tools such as Qualtrics (105) or Google Forms (106). To facilitate early and accurate collection of metadata about samples, only samples for which unique identifiers and a comprehensive set of metadata has been collected should be shipped to other sites. Data should only be shared when all the metadata is complete, they are uploaded to a repository (privately) or a common drive, and linked in the central database. Recipients can then use the unique identifiers to look up key information about each sample/dataset in the database and find all the necessary metadata. Access to the data before it is public can be decided as needed since considerations may vary depending on project needs or data type and source, eg: human versus non-human sequencing data.

To address the third point, establishing recurrent meetings that bringing together researchers of complementary expertise to discuss experimental design, data analysis, next steps, and synthesize information is important. This helps ensure that the data is analyzed and interpreted in a meaningful way, as well as used to inform the design of appropriate follow-up experiments.

## FAIR data sharing between interdisciplinary teams is critical to the responsible development and deployment of AI

To summarize, the life sciences are on the cusp of a transformation to a data-intense field that requires experimental and computational biologists to work together and make sense of large swaths of data using mechanistic, ML and DL models. This will allow researchers to generate new insights that drive biomedical research forward in ways and at a scale previous not possible. Enabling this involves embracing a collaborative and digital-first mentality to sustain the development of data hungry, unbiased, generalizable models that are helpful to biomedical researchers. We argue that the future of such a transformation involves a shift to the Digital Lab and decentralized FAIR data sharing and compute to enable broader collaboration across disciplines. Despite the complexity of the feat, it is necessary to ensure that data is scrutinized, used, and re-used to the best extent possible, maximizing return on investment in the research enterprise for the benefit of all.
